# Activation of a passive, mesoporous silica nanoparticle layer through attachment of bacterially-derived carbon-quantum-dots for protection and functional enhancement of probiotics

**DOI:** 10.1016/j.mtbio.2022.100293

**Published:** 2022-05-17

**Authors:** Hao Wei, Wei Geng, Xiao-Yu Yang, Jeroen Kuipers, Henny C. van der Mei, Henk J. Busscher

**Affiliations:** a University of Groningen and University Medical Center Groningen, Department of Biomedical Engineering, Antonius Deusinglaan 1, 9713 AV, Groningen, the Netherlands; b School of Chemical Engineering and Technology, Sun Yat-Sen University, Zhuhai, 519082, China; c Wuhan University of Technology, State Key Laboratory of Advanced Technology for Materials Synthesis and Processing & Shenzhen Research Institute of Wuhan University of Technology, Luoshi Road 122, 430070, Wuhan, China; d School of Engineering and Applied Sciences, Harvard University, Cambridge, MA, 02138, USA; e University of Groningen and University Medical Center Groningen, Department of Biomedical Sciences of Cells & Systems, Antonius Deusinglaan 1, 9713 AV, Groningen, the Netherlands

**Keywords:** Infection, Boron hydroxyl chemistry, Mesoporous nanoparticles, Probiotics, Food supplementation

## Abstract

Probiotic bacteria employed for food supplementation or probiotic-assisted antibiotic treatment suffer from passage through the acidic gastro-intestinal tract and unintended killing by antibiotics. Carbon-quantum-dots (CQDs) derived from bacteria can inherit different chemical groups and associated functionalities from their source bacteria. In order to yield simultaneous, passive protection and enhanced, active functionality, we attached CQDs pyrolytically carbonized at 220 ​°C from *Lactobacillus acidophilus* or *Escherichia coli* to a probiotic strain (*Bifidobacterium infantis*) using boron hydroxyl-modified, mesoporous silica nanoparticles as an intermediate encapsulating layer. Fourier-transform-infrared-spectroscopy, X-ray-photoelectron-spectroscopy and scanning-electron-microscopy were employed to demonstrate successful encapsulation of *B. infantis* by silica nanoparticles and subsequent attachment of bacterially-derived CQDs. Thus encapsulated *B. infantis* possessed a negative surface charge and survived exposure to simulated gastric fluid and antibiotics better than unencapsulated *B. infantis*. During *B. infantis* assisted antibiotic treatment of intestinal epithelial layers colonized by *E. coli*, encapsulated *B. infantis* adhered and survived in higher numbers on epithelial layers than *B. infantis* without encapsulation or encapsulated with only silica nanoparticles. Moreover, higher *E. coli* killing due to increased reactive-oxygen-species generation was observed. In conclusion, the active, protective encapsulation described enhanced the probiotic functionality of *B. infantis*, which might be considered as a first step towards a fully engineered, probiotic nanoparticle.

## Highlights

• *B. infantis* was encapsulated in silica nanoparticles, activated with bacterially-derived CQDs.
• Encapsulated *B. infantis* showed enhanced survival after exposure hostile conditions.
• CQD activation of encapsulated *B. infantis* enhanced ROS generation and pathogen killing.
• CQD activation of encapsulated *B. infantis* enhanced adhesion to intestinal epithelial layers.

## Graphical abstract

**Figure:**
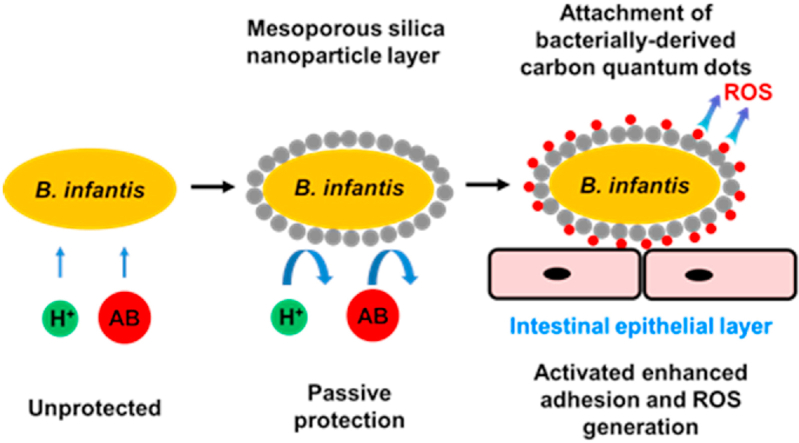

## 1 Introduction

Probiotic bacteria play an important role in maintaining a healthy intestinal microenvironment and restoration of a balanced microflora [[1], [2], [3]]. In addition, probiotic bacteria can assist antibiotic treatment of intestinal infection. However, viability of probiotic bacteria after oral administration can be severely hampered during passage through the acidic environment of the gastro-intestinal tract [4] as well as through unintended killing by antibiotics, supposed to kill only infecting pathogens [5]. Several passive, surface-engineered encapsulation methods have been developed in order to protect probiotic bacteria against harsh environmental conditions, such as gastric acids or antibiotics [[6], [7], [8]]. Encapsulation however, can negatively impact bacterial viability especially when the interaction between encapsulating molecules and bacterial cell surface components is strong, such as through coordinate-covalent bonding [9]. Alginate hydrogels bind weakly through hydrogen bonding [9]. Alginate hydrogel encapsulation protected probiotic lactobacilli against tobramycin while assisting tobramycin killing of pathogenic methicillin-resistant *Staphylococcus aureus* and *Pseudomonas aeruginosa* in co-culture [5]. Similarly, alginate-hydrogel encapsulated, probiotic *Bifidobacterium breve* assisted tetracycline killing of antibiotic-resistant *Escherichia coli* adhering to intestinal epithelial layers and maintained the barrier integrity of an epithelial cell layer [10]. Probiotic functionality includes a wide variety of mechanisms, including competitive adhesion and secretion of biosurfactants or reactive oxygen species (ROS) [11]. Especially secretion of biosurfactants across a protective encapsulating shell of is severely slowed down with a negative impact on probiotic killing of intestinal pathogens [9]. Therefore, alternative, more active encapsulation methods for the protection of probiotic bacteria are required that simultaneously maintain or enhance important probiotic functionalities for pathogen killing. To this point, bulk encapsulation of multiple bacteria has recently been discouraged in comparison with single-cell encapsulation [[12], [13], [14]]. Furthermore, active encapsulation of single cells has been advocated to restore possibly hampered functionalities after passive encapsulation. In active encapsulation, encapsulating shells are equipped with advanced features to enhance endogenous reactions of cells or stimulating (bio)chemical reactions that cannot be achieved by the encapsulated cells themselves [14].

Carbon quantum dots (CQDs) are nanoparticles with extremely small size that can be derived from carbonization of suitable carbon sources, amongst which bacteria. Recently it has been shown that CQDs synthesized by pyrolytic carbonization of bacteria at intermediate temperatures between 200 ​°C and 220 ​°C inherit amide groups and nitrogen hetero-atoms from their source bacteria [15]. This chemical inheritance was accompanied by more extensive generation of ROS. CQDs derived from bacteria exhibited up to 100-fold higher generation of ROS than their source bacteria due to hetero-atoms in CQDs that increase free electron incorporation in carbon dots and therewith ROS generation [15]. Based on these observations, we here forward the hypothesis that attachment of CQDs derived from bacteria to a passive, protective surface-engineered shell may actively enhance the functionality of encapsulated, probiotic bacteria while maximally maintaining viability of the encapsulated bacteria.

**Fig. 1:**
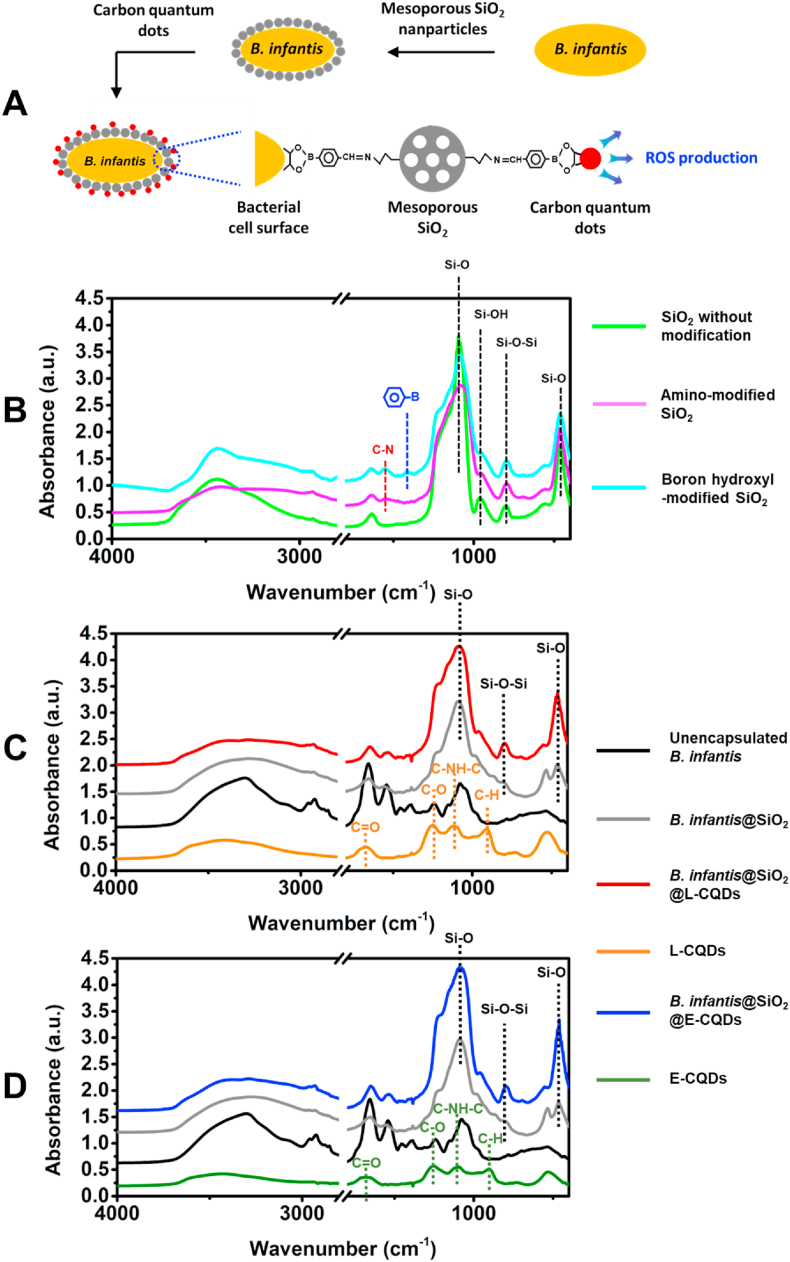
*B. infantis* ATCC 15697 encapsulation in a boron hydroxyl-modified, mesoporous silica nanoparticle layer with attached bacterially-derived CQDs. **(A)** Schematics of the encapsulation of bacteria in a passive shell of mesoporous silica nanoparticles, followed by activation of the shell through attachment of bacterially-derived CQDs. **(B)** FTIR absorption spectra of mesoporous silica nanoparticles, prior to and after amino- and subsequent boron hydroxyl-modification. Characteristic absorption peaks due to silica and amino groups are indicated by black- and red-dashed lines respectively, while the blue-dashed line points to an absorption peak at 1412 ​cm^−1^ due to boron directly coupled to an aromatic ring. **(C)** FTIR absorption spectra of unencapsulated *B. infantis*, *B. infantis* encapsulated in a silica nanoparticle layer in absence (*B. infantis*@SiO_2_) or presence of attached CQDs, derived from *L. acidophilus* ATCC 4356 (L-CQDs) and the L-CQDs. **(D)** Same as panel C, now for CQDs derived from *E. coli* ATCC 25922 (E-CQDs).

The aim of this paper is to verify this hypothesis by encapsulating probiotic *Bifidobacterium infantis* with CQDs derived from pyrolytically carbonized *Lactobacillus acidophilus* or *E. coli*. To this end, *B. infantis* was first encapsulated with a passive layer of boron hydroxyl-modified, mesoporous silica nanoparticles [16] after which click-chemistry was applied to attach bacterially-derived CQDs to the layer (see Fig. 1A for schematics). Physico-chemical properties of unencapsulated and encapsulated *B. infantis* as well as the protection offered by encapsulation against simulated gastric acid and tetracycline were determined. Probiotic activities were compared by co-culturing encapsulated *B. infantis* with *E. coli,* while adhesion and survival of encapsulated *B. infantis* to human intestinal epithelial cell layers was measured in a transwell model.

## 2 Materials and methods

### 2.1 Mesoporous silica nanoparticles synthesis and modification

Mesoporous silica nanoparticles were synthesized and modified, as described before [16]. Cetyltrimethylammonium (10.4 ​g) chloride solution (CTAC, Sigma-Aldrich, USA) and diethanolamine (0.2 ​g, DEA, Sigma-Aldrich, USA) were dissolved in demineralized water (64 ​mL) and ethanol (11.4 ​mL, 100%), under stirring in an oil bath at 60 ​°C. After 30 ​min, tetraethyl orthosilicate (7.3 ​mL, TEOS, Sigma-Aldrich, USA) was added under stirring and kept for 3 ​h at 60 ​°C. Next, silica nanoparticles in suspension were washed by demineralized water for three times by centrifugation (7000 ​g, 5 ​min), resuspended in water (5 ​mL), collected by freeze-drying (Leybold, Germany) and refluxed overnight in a solution of ethanol (120 ​mL, 96%) and hydrochloric acid (30 ​mL, 36–38%, Merck KGaA, Germany) to extract surfactants. Mesoporous silica nanoparticles were washed three times with demineralized water by centrifugation (7000 ​g, 5 ​min) and resuspended in demineralized water (5 ​mL). Finally, the suspension was freeze-dried to collect the silica nanoparticles.

For amino-modification of mesoporous silica nanoparticles, mesoporous silica nanoparticles (0.2 ​g) were suspended in anhydrous toluene (100 ​mL, Biosolve BV, The Netherlands) and 3-aminopropyltriethoxysilane (0.15 ​mL, APTES, Sigma-Aldrich, USA) was added. The suspension was refluxed overnight at 70 ​°C. Finally, the amino-modified mesoporous silica nanoparticles were washed three times in ethanol (96%) by centrifugation (7000 ​g, 5 ​min) and suspended in demineralized water, followed by freeze-drying for collecting the nanoparticles.

For boron hydroxyl-modification of amino-modified, mesoporous silica nanoparticles, amino-modified silica nanoparticles (0.1 ​g) and 4-formylphenylboronic acid (0.1 ​g, Sigma-Aldrich, USA) were mixed in anhydrous toluene (100 ​mL) after which glacial acetic acid (100 ​μL) was added dropwise and mixed under stirring for 5 ​min. The suspension was refluxed overnight at 70 ​°C, after which the boron hydroxyl-modified, mesoporous silica nanoparticles were washed three times by ethanol (96%) (centrifugation, 7000*g*, 5 ​min) and re-suspended in demineralized water, followed by freeze-drying for collecting the nanoparticles.

### 2.2 Bacterial growth and harvesting

*B. infantis* ATCC 15697 was grown on RCM agar plates (Reinforced Clostridial Medium, Becton, Dickinson and Company, France) under anaerobic conditions (85% N_2_, 10% H_2_, 5% CO_2_, at 37 ​°C) for 48 ​h [9]. Then, a single colony was picked from the agar plate and suspended in RCM (10 ​mL) broth and cultured under anaerobic conditions. After 24 ​h, the pre-culture was transferred into RCM (200 ​mL) broth and cultured under the same conditions for 18 ​h. After 18 ​h, bacteria were harvested by centrifugation (5000 ​g, 5 ​min, 10 ​°C) and washed with 10 ​mM potassium phosphate buffer (pH 7.0) for three times and re-suspended in 10 ​mM phosphate buffer (5 ​mL). The bacterial suspension was sonicated at 130 ​W for 30 ​s to disrupt possible aggregates. Bacterial concentration was determined in a Bürker-Türk counting chamber and fixed at 3 ​× ​10^10^ ​mL^−1^ for *B. infantis*. *L. acidophilus* ATCC 4356 was grown on MRS (De Man, Rogosa and Sharpe) agar plates in 5% CO_2_ at 37 ​°C for 48 ​h. Then, a single colony was picked from the agar plate and suspended in MRS broth (10 ​mL and cultured under anaerobic conditions at 37 ​°C. After 24 ​h, the pre-culture was transferred into MRS broth (200 ​mL) and cultured under the same conditions for 18 ​h. After 18 ​h, bacteria were harvested by centrifugation (5000 ​g, 5 ​min, 10 ​°C) and washed with demineralized water for two times. The bacterial pellets were collected in ceramic crucibles for further synthesis of carbon quantum dots (CQDs). *E. coli* ATCC 25922 were cultured as described for *L. acidophilus*, but using BHI (brain heart infusion) agar or BHI broth (Oxoid Ltd, UK) under aerobic conditions.

### 2.3 Bacterially-derived carbon quantum dots

Bacterially-derived CQDs were synthesized, as described before [15]. Briefly, bacterial pellets of *L. acidophilus* or *E. coli* suspension were heated in ceramic crucibles at a rate of 5 ​°C min^−1^ to 220 ​°C and kept for 2 ​h at 220 ​°C. After 2 ​h, the crucibles containing the reaction product were cooled down to room temperature. Reaction products were dispersed in demineralized water and sonicated for 2 ​h. For purification and obtaining suspensions with mono-disperse CQDs, suspensions were filtered through a 0.22 ​μm polyvinylidene difluoride filter membrane, followed by dialysis for 48 ​h against demineralized water (cut-off 1 ​kDa) under stirring at room temperature. Finally, CQDs suspended in water were collected by freeze-drying. Quantum yields of thus derived CQDs were shown by analysis of their integrated fluorescence intensity as a function of absorbance, to be 1.0% and 1.5% relative to quinine sulfate for *L. acidophilus* and *E. coli*, respectively [15].

### 2.4 Encapsulation of B. infantis by mesoporous silica nanoparticles and attachment of CQDs

For bacterial encapsulation in a mesoporous silica nanoparticle layer, boron hydroxyl-modified silica nanoparticles were added into 10 ​mM potassium phosphate buffer (10 ​mL) to a final concentration of 1 ​mg ​mL^−1^ and sonicated for 5 ​min. Then, *B. infantis* was added into the suspension (10 ​mL) at a final concentration of 3 ​× ​10^8^ ​mL^−1^, vortexed for 1 ​min and allowed to settle at room temperature for 15 ​min. Finally, encapsulated *B. infantis* were collected by centrifugation (5000 ​g, 5 ​min, 10 ​°C) for attachment of bacterially-derived carbon quantum dots.

To this end, silica nanoparticle encapsulated *B. infantis* were added into 10 ​mM potassium phosphate buffer containing CQDs (0.5 ​mg ​mL^−1^) derived from *L. acidophilus* or *E. coli* and re-suspended by vortexing for 1 ​min. Suspensions were kept at room temperature for 15 ​min to allow attachment of CQDs. Subsequently, encapsulated *B. infantis* were harvested by centrifugation (5000 ​g, 5 ​min, 10 ​°C) and washed by 10 ​mM phosphate buffer to remove non-attached CQDs. In a selected number of experiments, CQDs were directly attached to the *B. infantis* cell surface without an intermediate silica nanoparticle layer. This was done similarly as described above, but now adding unencapsulated *B. infantis* into 10 ​mM potassium phosphate buffer containing CQDs (0.5 ​mg ​mL^−1^) derived from *L. acidophilus* or *E. coli*. The suspension was kept at room temperature for 15 ​min to allow attachment of CQDs. Subsequently, bacteria with directly attached CQDs were harvested by centrifugation and re-suspended in 10 ​mM phosphate buffer by vortexing for 1 ​min.

### 2.5 Viability of unencapsulated and encapsulated B. infantis

Agar plating and CFU enumeration were applied to determine the viability of unencapsulated and encapsulated *B. infantis*. Bacteria were collected by centrifugation (5000 ​g, 5 ​min, 10 ​°C) and re-suspended by vortexing in phosphate buffer (10 ​mM). Serial dilutions of the bacterial suspensions were made in 10 ​mM phosphate buffer and plated on RCM agar plates. After 48 ​h culturing at 37 ​°C in an anaerobic chamber, the numbers of CFUs were enumerated.

### 2.6 Fourier transform infrared spectroscopy (FTIR)

FTIR spectra were measured on an Agilent Cary 600 series FTIR spectrometer (Agilent Technologies, USA). Freeze-dried unencapsulated and encapsulated *B. infantis*, bacterially-derived CQDs and unmodified and modified SiO_2_ were mixed with KBr powder (100 ​mg KBr with 2% w/w of sample) and pressed into a tablet for measurements. FTIR spectra were recorded over the wavenumber range of 4000 and 400 ​cm^−1^ with a resolution of 4 ​cm^−1^.32 scans were measured and averaged. A KBr pellet without bacteria was used as background.

### 2.7 X-ray photoelectron spectroscopy (XPS)

XPS was performed with an S-Probe instrument (Surface Science Instrument, USA) with an Al-anode (1486 ​eV). To this end, unencapsulated or encapsulated *B. infantis* suspended in demineralized water were freeze-dried (Leybold, Germany) for 48 ​h. Prior to freeze-drying, both unencapsulated and encapsulated bacteria were extensively washed in water to remove remnants of medium or buffer components. The freeze-dried bacterial powders obtained were pressed into small stainless-steel cups, and put into the XPS chamber, while CQDs were deposited on gold-coated glass slides and put in the XPS chamber. X-ray production occurred by means of a magnesium anode (10 ​kV, 22 ​mA) using a spot size of 250 ​μm ​× ​1000 ​μm. Scans were made of the overall spectrum in the binding energy range of 1–1200 ​eV at low resolution, setting the C_1s_ binding energy at 284.8 ​eV. The area under each peak in overall spectra, after background subtraction, was used for the calculation of peak intensities, yielding elemental surface concentrations. High resolution, narrow scans were recorded over a 20 ​eV binding energy range of the N_1s_ peak. The N_1s_ peak was decomposed into two Gaussian peak components due to proteinaceous C–NH_2_ at 399.8 ​eV and due to C–NH_3_^+^ at 401.3 ​eV, each with a full width at half maximum of 1.6 ​eV.

### 2.8 Zeta potential measurements

Zeta potentials were measured in a Malvern ZetaSizer (model: ZEN3600, serial nr: MAL1037113) in 10 ​mM potassium phosphate buffer with pH adjusted to range between 2 and 9 using a PHM220 laboratory pH meter (Radiometer Analytical SAS, France). pH was adjusted by adding a solution of HCl (0.1 ​M) or KOH (0.1 ​M). To this end, unencapsulated or encapsulated *B. infantis* were suspended in potassium phosphate buffer with different pH to yield a concentration of 3 ​× ​10^7^ ​mL^−1^. For measurement of the zeta potentials of bacterially-derived CQDs, CQDs were suspended at a concentration of 0.1 ​mg ​mL^−1^ in potassium phosphate buffer.

### 2.9 Scanning electron microscopy (SEM)

SEM micrographs were taken using a Supra 55 Scanning Electron Microscope (Zeiss) at an acceleration voltage of 3 ​kV and a working distance of 7.5 ​mm. For sample preparation, unencapsulated and encapsulated *B. infantis* were pelleted by centrifugation (5000 ​g, 5 ​min, 10 ​°C) and resuspended in a solution of paraformaldehyde (2%) and glutaraldehyde (2%) in cacodylate buffer for fixation. After fixation for 30 ​min, the bacterial suspension (100 ​μL) was dropped on a poly-L-lysine coated glass coverslip. Poly-L-lysine coated coverslips were prepared by dropping poly-L-lysine (1 ​mg ​mL^−1^) on a glass coverslip, drying at 37 ​°C in an oven for 30 ​min and triple washing in phosphate buffered saline (PBS, 10 ​mM potassium phosphate buffer and 0.15 ​M NaCl, pH 7.4). After dropping the bacterial suspension on the coverslip and allowing bacterial sedimentation for 30 ​min, the coverslip was washed twice by PBS and once with cacodylate buffer to remove non-adhering bacteria. Then, OsO_4_ (1%) solution in cacodylate buffer was used to fix bacteria for 1 ​h, after which bacteria were washed with ultrapure water for 3 times. Fixed bacteria were dehydrated with 30, 50, 70% ethanol for 15 ​min each and 100% ethanol, 3 times for 30 ​min each. Finally, samples were incubated in an ethanol (100%)/tetramethylsilane (TMS) 1:1 solution for 10 ​min, followed by immersion for 15 ​min in pure TMS and air dried.

### 2.10 Porosity measurements

Nitrogen adsorption/desorption isotherms of unencapsulated and encapsulated *B. infantis* were determined to derive the BET surface area (Brunauer-Emmett-Teller) and pore diameters of the shells using a Tristar instrument (Micromeritics, USA). First, unencapsulated or encapsulated *B. infantis* were suspended in demineralized water and subsequently freeze-dried. Lyophilized bacteria were further degassed at 40 ​°C for 12 ​h and measurements were performed in liquid nitrogen (−196 ​°C). BET surface areas were derived by fitting the adsorption/desorption isotherms to

(1) $$\frac{1}{\left[V_{a}\left(\frac{P_{0}}{P}-1\right)\right]}=\frac{C-1}{V_{m}C}\times \frac{P}{P_{0}}+\frac{1}{V_{m}C}$$

(2) $$S=\frac{V_{m}N_{a}}{m\times 22400}$$

in which *V*_*ɑ*_ is volume of gas adsorbed at standard temperature and pressure (STP, i.e. 0 °C and 1.013 ​× ​10^5^ ​Pa), *V*_*m*_ is volume of gas adsorbed at STP to produce an apparent monolayer on the sample surface, *P*_*0*_ is saturated pressure of adsorbate gas, *P* is partial vapor pressure of adsorbate gas in equilibrium with the surface at −196 ​°C, *C* is a dimensionless constant related to the enthalpy of adsorption of the adsorbate gas on the sample, *S* is BET surface area, *N*_*ɑ*_ is Avogadro constant and *m* is the mass of the sample. The shape of pores was assumed to be cylindrical and average pore diameters were calculated accordingly using

(3) $$d=\frac{4V}{S}$$

in which *d* is average pore diameter, *V* is the total pore volume and *S* is BET surface area.

### 2.11 Protection against simulated gastric fluid

Simulated Gastric Fluid was prepared as published previously [10]. Briefly, NaCl (2.0 ​g, Merck KGaA, Germany) and pepsin (6.0 ​g, porcine, Sigma-Aldrich, USA) were dissolved in hydrochloric acid (7 ​mL, 36–38%). The solution was diluted with demineralized water to 1 ​L after which pH was adjusted to pH 3 with NaOH (1 ​M). For evaluating the protection offered by the shells, 3 ​× ​10^8^ ​mL^−1^ unencapsulated or encapsulated *B. infantis* were exposed to simulated gastric fluid. After shaking at 37 ​°C (150 ​rpm) for 30 ​min and 60 ​min, bacteria were collected by centrifugation (5000 ​g, 5 ​min, 10 ​°C), washed twice with phosphate buffer (10 ​mM) and their viability was assessed by plate counting on RCM agar plates.

### 2.12 Probiotic activity of unencapsulated and encapsulated B. infantis

Probiotic activity of unencapsulated and encapsulated *B. infantis* were determined in a planktonic co-culture model. In the co-culture model, 3 ​× ​10^9^ ​mL^−1^ unencapsulated or encapsulated *B. infantis* with or without pre-exposure to pH 3 simulated gastric fluid for 30 ​min were co-cultured for 24 ​h with 3 ​× ​10^4^ ​mL^−1^ ​*E. coli* in RCM medium in an anaerobic chamber at 37 ​°C. After that, the number of viable *E. coli* and *B. infantis* in the co-culture medium were enumerated by plating on BHI or RCM agar plates, respectively.

### 2.13 ROS generation

ROS generation by unencapsulated or encapsulated *B. infantis* was determined by oxidation of non-fluorescent 2′,7′-dichlorohydrofluorescin (DCFH) to highly fluorescent 2′,7′-dichlorofluorescein (DCF) [17]. DCFH was prepared by 30 ​min hydrolysis of 10 ​mg ​mL^−1^ 2′,7′-dichlorohydrofluorescein diacetate (DCFDA), dissolved in dimethylsulfoxide) in the presence of NaOH (0.01 ​M) in the dark at room temperature. Next, the mixture was neutralized with potassium phosphate buffer (10 ​mM, pH 7.4) to obtain a final concentration of 10 ​μg ​mL^−1^ DCFH. Freshly prepared bacterial suspensions (200 ​μL, 1 ​× ​10^8^ bacteria per mL) were put in 96-wells plates, 10 ​μL DCFH was added to each well and plates were kept in the dark for 1 ​h at 37 ​°C. After 1 ​h, fluorescence intensities were measured at excitation and emission wavelengths of 485 and 528 ​nm, respectively. Fluorescence intensity of DCFH dissolved in potassium phosphate buffer without bacteria amounted 4.1 ​± ​0.9 (arbitrary units).

### 2.14 B. infantis-assisted antibiotic-treatment of intestinal epithelial layers colonized by E. coli

Human intestinal epithelial cells (Caco-2 BBe, ATCC CRL-2102) were prepared in a transwell model, as described before [10]. Cells were first grown in DMEM-HG (Thermo Fisher Scientific, USA) supplemented with fetal bovine serum (10% (v/v), FBS, Thermo Fisher Scientific, USA) in 5% CO_2_ humidified air at 37 ​°C and passaged at 80% confluency after trypsinization for 5 ​min using trypsin−EDTA at 37 ​°C. After detachment, 6 ​mL DMEM-HG supplemented with FBS (10%) was added for trypsin neutralization and cells were collected by centrifugation at 800*g* for 5 ​min. The cellular pellet was re-suspended in DMEM-HG supplemented with FBS (10%), and cell concentration was adjusted to 2 ​× ​10^5^ ​cells mL^−1^ by counting in a Burker-Turk counting chamber. Then, 0.5 ​mL cell suspension was added to a transwell insert in a 12-well plate equipped with a 1.13 ​cm^2^ PET membrane (pore size 0.4 ​μm). During growth of the cellular layer, the medium was refreshed every other day.

After culturing for 10 days, 50 ​μL of an *E. coli* suspension in PBS (3 ​× ​10^7^ ​mL^−1^) was added on the intestinal epithelial cell layer and co-cultured for 2 ​h with epithelial cells in co-culture medium [18] (70% DMEM-HG with 30% RCM) in 5% CO_2_ at 37 ​°C. After 2 ​h, co-culture medium was refreshed to remove unattached *E. coli*. Next, unencapsulated or encapsulated *B. infantis* was pre-exposed to simulated gastric fluid (pH 3) for 30 ​min, after which bacteria were suspended in PBS (3 ​× ​10^8^ ​mL^−1^) and added to the epithelial cell layers. PBS without *B. infantis* was used as a control. Simultaneously, tetracycline was added to a final concentration of 10 ​μg ​mL^−1^. After 3 ​h incubation, cell layers with adhering bacteria were washed twice with PBS and detached using trypsin−EDTA (500 ​μL, 2.5 ​mg ​mL^−1^, 5 ​min at 37 ​°C). The resulting suspension was centrifuged and washed twice in PBS (5000 ​g, 5 ​min) to remove the trypsin and finally re-suspended in 0.5 ​mL PBS by vortexing for 1 ​min. After serial dilution, the number of viable *E. coli* and *B. infantis* in the suspension were enumerated by plating on BHI or RCM agar plates, respectively as described above for planktonic co-cultures.

In separate experiments, the cell surface coverage of the transwell membranes by epithelial cells after co-culturing was determined by staining with phalloidin-FITC (F-actin) and DAPI (DNA). Briefly, cell layers with adhering bacteria were washed with PBS buffer for 5 ​min, fixed with paraformaldehyde (3.7% (w/v)) for 15 ​min, and permeabilized with Triton X-100 (0.5% (v/v)) for another 5 ​min. Subsequently, epithelial cells were stained with phalloidin-FITC (diluted 50 ​× ​in PBS with 1% (w/v) bovine serum albumin) and DAPI (diluted 50 ​× ​in PBS with 1% (w/v) bovine serum albumin) for 30 ​min and washed with PBS. The cells were imaged using fluorescence microscopy (Leica DM4000, Germany) and surface coverage was determined using Fiji software. Five images per sample were averaged.

### 2.15 Statistical analysis

All experiments were done in triplicate with separately cultured and encapsulated bacteria. Results were shown as mean ​± ​standard deviation and statistically significant differences (p ​< ​0.05) were determined using a Student t-test.

## 3 Results

### 3.1 Physico-chemical properties of B. infantis encapsulated with bacterially-derived CQDs

The first step in the encapsulation of *B. infantis* with bacterially-derived CQDs is the encapsulation of *B. infantis* inside a layer of mesoporous silica nanoparticles. To this end, silica nanoparticles were first amino-modified using APTES to covalently couple aminopropyl groups directly onto the surface of silica nanoparticles. Amino-modification of silica nanoparticles yielded a new absorption peak at 1546 ​cm^−1^ due to C–N [19] in the Fourier transform infrared (FTIR) spectrum of silica nanoparticles, while subsequent boron hydroxyl-modification, achieved by reaction of amino groups with 4-formylphenylboronic acid, yielded a new absorption peak at 1412 ​cm^−1^ due to boron directly coupled to an aromatic ring [20] (Fig. 1B).

Encapsulation of *B. infantis* with mesoporous silica nanoparticles is accompanied by the development of new absorption peaks at 465 ​cm^−1^ (Si–O), 790 ​cm^−1^ (Si–*O*–Si), 964 ​cm^−1^ (Si–OH) and 1093 ​cm^−1^ (Si–O), absent in the spectrum of unencapsulated *B. infantis* (Fig. 1C and **D**). Bacterially-derived CQDs from *L. acidophilus* (Fig. 1C) and *E. coli* (Fig. 1D) possess similar absorption peaks, most notably at 900 ​cm^−1^ due to aromatic C–H [21], at 1120 ​cm^−1^ due to C–NH–C, at 1240 ​cm^−1^ due to C–O and at 1680 ​cm^−1^ due to C=O [22] that collectively reflect the inheritance of amide functionalities from bacterial proteins and aromatic carbons from bacterial hydrocarbon-like surface components after pyrolytic carbonization.

For further characterization of the encapsulating shells, X-ray photoelectron spectroscopy (XPS) was applied. Surface elemental compositions of differently encapsulated *B. infantis* after freeze-drying are presented in Table 1. The surface of unencapsulated *B. infantis* was mainly composed of carbon, nitrogen, oxygen and phosphorus. In an interpretative model [23], these data could be attributed to protein (11%), polysaccharide (68%), hydrocarbon-like compounds (15%) and teichoic acid (7%). After encapsulation of *B. infantis* by boron hydroxyl-modified mesoporous silica nanoparticles, the nitrogen surface concentration increased from 1.9 ​at% to 2.8 ​at%, while Si was clearly detected (13.6 ​at%). Both N and Si are due to the presence of amino-modified silica nanoparticles, although N involved in proteinaceous C–NH_2_ groups (N_399.8 ​eV_, see Table S1) remained similarly low (2.1 ​at%) after encapsulation by silica nanoparticles (1.8 ​at%). Si remained to be detected in similarly high surface concentrations after attachment of bacterially-derived CQDs. This attests to the small size of the attached CQDs, unable to attenuate the photoelectron flux from the underlying silica nanoparticles. This is furthermore supported by only minor increases in the amount of proteinaceous C–NH_2_ groups (N_399.8 ​eV_) after attachment of bacterially-derived CQDs, even when protein-rich CQDs derived from protein-rich lactobacilli were involved (Table S1). *B. infantis* surfaces possessed very little P (0.4 ​at%, see Table 1). P surface concentration increased five-fold upon encapsulation with a mesoporous silica nanoparticle layer, likely because encapsulation was done in a phosphate buffer and phosphate remnants were maintained in the mesoporous silica nanoparticle layer, despite washing with demineralized water prior to XPS examination. *L. acidophilus* derived CQDs possessed a larger surface concentration of P than *E. coli* derived CQDs, suggesting presence of phosphate groups as an inheritance from their source bacteria. These differences did not show in the surface concentration of P upon attachment to the mesoporous silica nanoparticle layer, pointing to a low number of attached CQDs.

**Table 1:** Elemental surface compositions of unencapsulated *B. infantis*, *B. infantis* encapsulated in a mesoporous silica nanoparticle layer in absence (*B. infantis*@SiO_2_) or presence of attached, bacterially-derived CQDs, obtained by XPS.

Samples	Elemental surface compositions (at%)^a^				
	C	N	O	P	Si
Unencapsulated *B. infantis*	61.5	1.9	35.8	0.4	−a
*B. infantis*@SiO_2_	38.9	2.8	34.9	2.0	13.6
*B. infantis*@SiO_2_@L-CQDs	33.1	2.9	36.5	2.1	14.5
L-CQDs	35.4	5.4	39.1	6.2	−a
*B. infantis*@SiO_2_@E-CQDs	36.5	3.5	33.7	2.7	12.3
E-CQDs	46.5	3.0	33.4	2.1	−a

a Below detection.

**Fig. 2:**
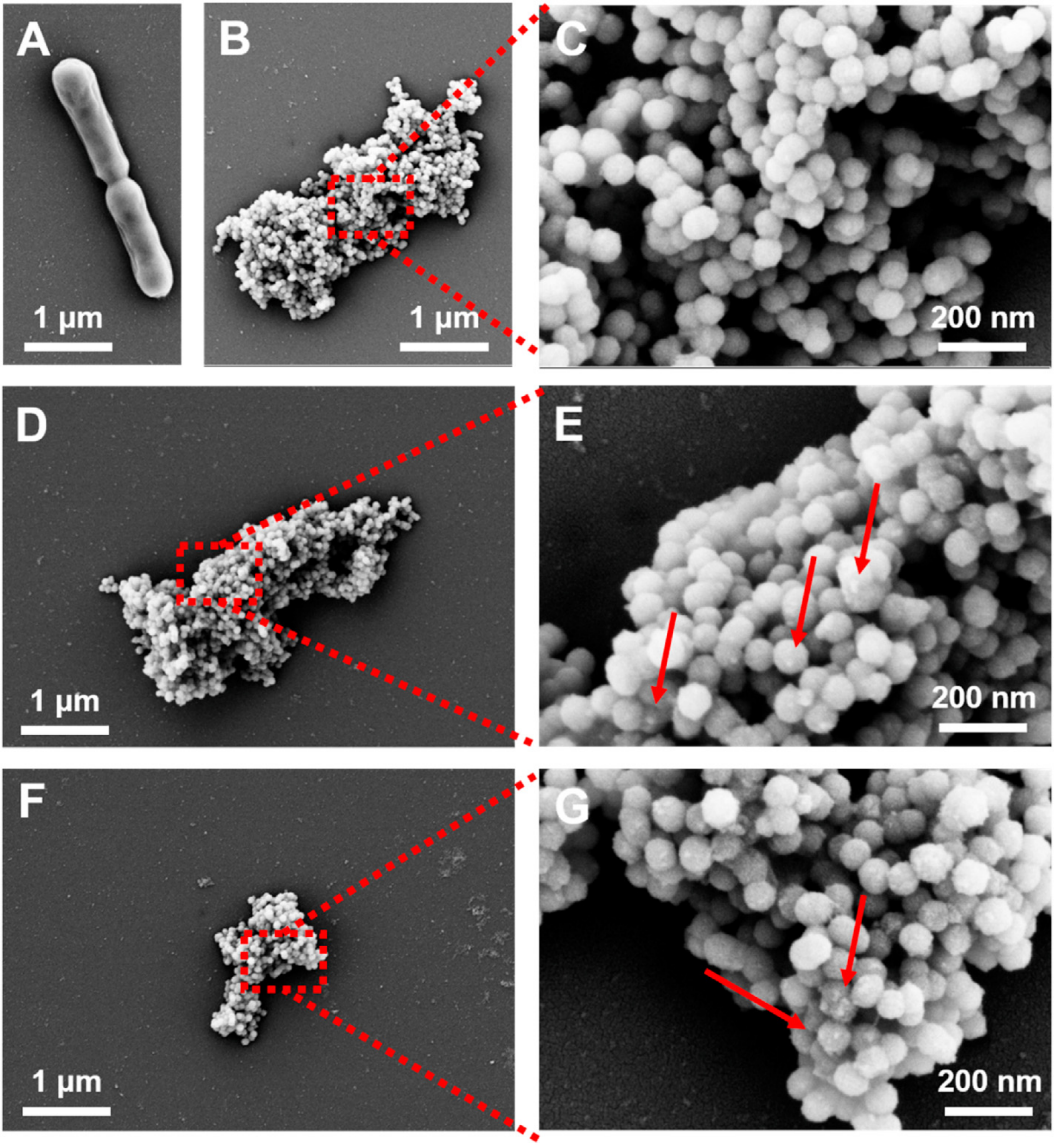
SEM micrographs of differently encapsulated *B. infantis* ATCC 15697. **(A)** Micrograph of unencapsulated *B. infantis*. **(B)** Micrograph of *B. infantis* encapsulated in a mesoporous silica nanoparticle layer. **(C)** Micrograph of a selected area in panel B, indicated by the red square. **(D)** Micrograph of *B. infantis* encapsulated in a mesoporous silica nanoparticle layer, with subsequent attachment of CQDs derived from *L. acidophilus* ATCC 4356. **(E)** Micrograph of a selected area in panel D, indicated by the red square. Bright dots, indicated by arrows, point to attached CQDs. **(F)** Same as panel D, now with subsequent attachment of CQDs derived from *E. coli* ATCC 25922. **(G)** Micrograph of a selected area in panel F, indicated by the red square. Bright dots, indicated by arrows, point to attached CQDs.

Encapsulation of *B. infantis* by mesoporous silica nanoparticles can be clearly observed electron microscopically (compare SEM micrographs of unencapsulated and nanoparticle encapsulated *B. infantis* (Fig. 2A and Fig. 2B, respectively). Subsequent attachment of bacterially-derived CQDs did not affect the structure of the silica layer. Attached CQDs derived from *L. acidophilus* and *E. coli* appeared as scarcely distributed bright dots in high magnification SEM micrographs (compare Fig. 2D and **E** and Fig. 2F and **G,** respectively). Such bright dots were not visible in high magnification micrographs of *B. infantis* only encapsulated by silica nanoparticles without attached bacterially-derived CQDs (Fig. 2C). The scarce distribution of CQDs on the silica nanoparticles confirms attachment of CQDs in low numbers to the mesoporous silica nanoparticle layer, although presence of CQDs inside the mesoporous layer cannot be ruled out. This is in line with the suggestion of low-number attachment evidenced by XPS data.

**Fig. 3:**
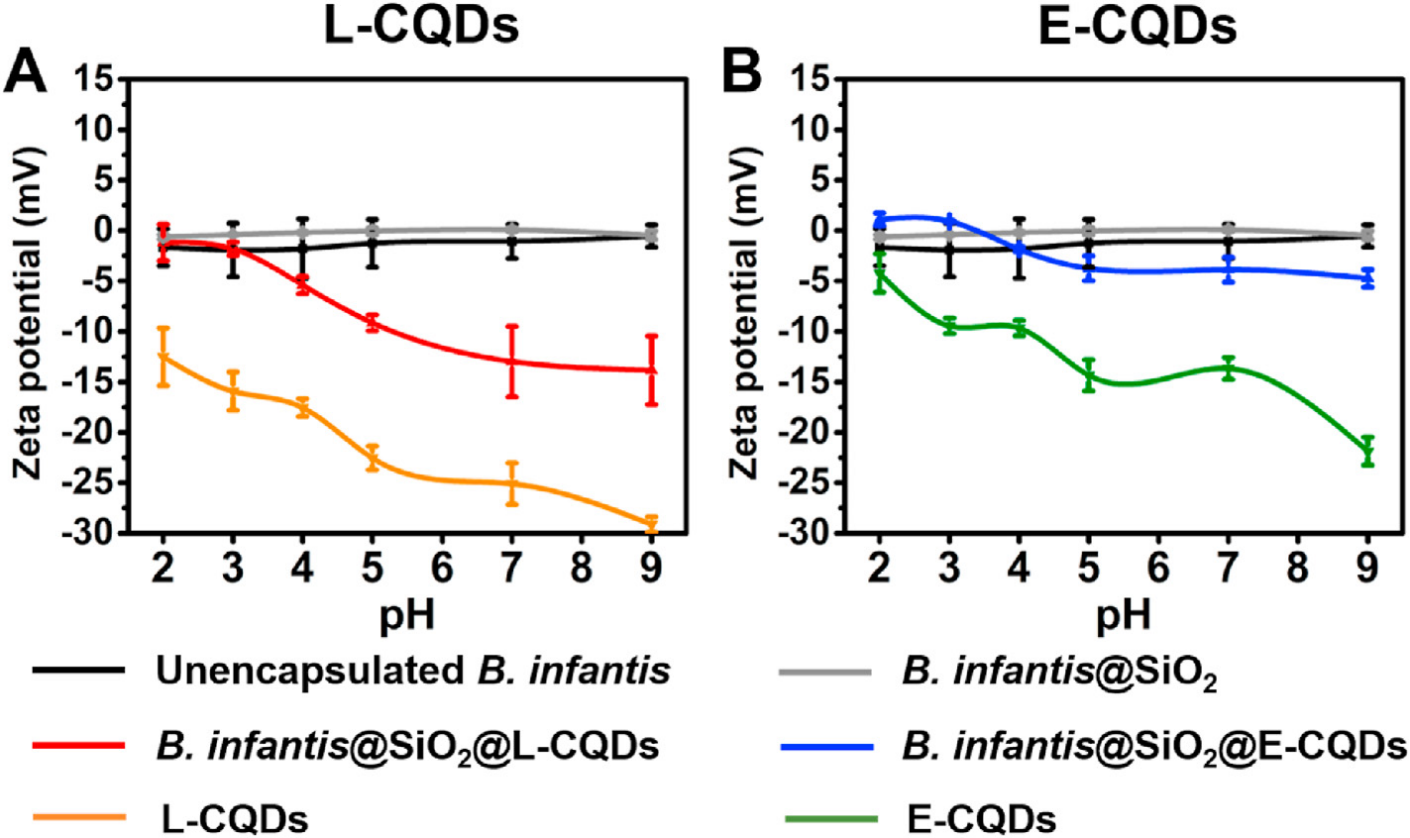
Zeta potentials of differently encapsulated *B. infantis* ATCC 15697 and bacterially-derived CQDs in 10 ​mM phosphate buffer. **(A)** Zeta potentials of unencapsulated *B. infantis*, *B. infantis* encapsulated in a mesoporous silica nanoparticle layer in absence (*B. infantis*@SiO_2_) or presence of attached *L. acidophilus*-derived L-CQDs. **(B)** Same as panel A, now for attached CQDs derived from *E. coli* ATCC 25922 (E-CQDs). All error bars indicate standard deviations over triplicate experiments with separately cultured bacteria.

Zeta potentials of *B. infantis* hovered around 0 ​mV across the entire pH range from 2 to 9 (Fig. 3). Encapsulation in a layer of mesoporous silica nanoparticles did not affect bacterial zeta potentials. This is probably because the mesoporous nanoparticle layer constituted a soft layer, through which micro-electrophoretic fluid flow can penetrate to bring the plane of shear close to the bacterial cell wall [24], resulting in the measurement of near-identical zeta potentials as measured for unencapsulated *B. infantis*. Upon attachment of bacterially-derived CQDs to the encapsulating nanoparticle layers, zeta potentials became more negative. Arguably, effects of CQD attachment are too large to be due to the relatively few CQDs attached to the outside of the mesoporous nanoparticle layer (Fig. 2). Taken together with the soft layer character of the layer, this supports our previous suggestion that CQDs must also present inside the encapsulating layer. Accordingly, attachment of *L. acidophilus* derived CQDs yielded more negative zeta potentials (Fig. 3A) than attachment of *E. coli* derived CQDs (Fig. 3B), alike *L. acidophilus*-derived CQDs being more negatively charged than *E. coli* CQDs (see also Fig. 3). The negative zeta potentials of both types of CQDs over the entire pH range confirm the suggestion based on elemental surface compositions that P is due to phosphate groups that carry a low iso-electric point [25]. Moreover, the more negative zeta potentials of L-CQDs as compared with E-CQDs are in line with the higher elemental surface concentration of P in L-CQDs (Table 1).

Collectively, the above data yield the conclusion that boron hydroxyl-modified, mesoporous silica nanoparticles can be successfully encapsulated on the surface of *B. infantis*. The additional attachment of bacterially-derived CQDs yielded scattered CQDs on the surface of the mesoporous silica nanoparticle layer as well as inside the layer.

Nitrogen adsorption-desorption isotherms of differently encapsulated *B. infantis* (Fig. S1) were analyzed according to Brunauer–Emmett–Teller (BET), yielding BET surface areas for *B. infantis* after different encapsulations between 34 and 39 ​m^2^ ​g^−1^ and pore diameters of 20–23 ​nm (Table 2). This indicates that the mesoporous silica layer is the main contributing factor to the porosity of the shells. Additional attachment of bacterially-derived CQDs did not yield significant blocking of the pores, because the CQDs had considerably smaller diameters (2–3 ​nm) [15] than the pores.

**Table 2:** BET surface areas and pore diameters of *B. infantis* ATCC 15697 encapsulated in a mesoporous silica nanoparticle layer in absence (*B. infantis*@SiO_2_) or presence of attached *L. acidophilus* ATCC 4356-derived (L-CQDs) or *E. coli* ATCC 25922-derived CQDs (E-CQDs).

Samples	Surface area (m^2^ g^−1^)	Pore diameter (nm)
*B. infantis*@SiO_2_	39	20
*B. infantis*@SiO_2_@L-CQDs	35	22
*B. infantis*@SiO_2_@E-CQDs	34	23

### 3.2 Bacterial viability after encapsulation

**Fig. 4:**
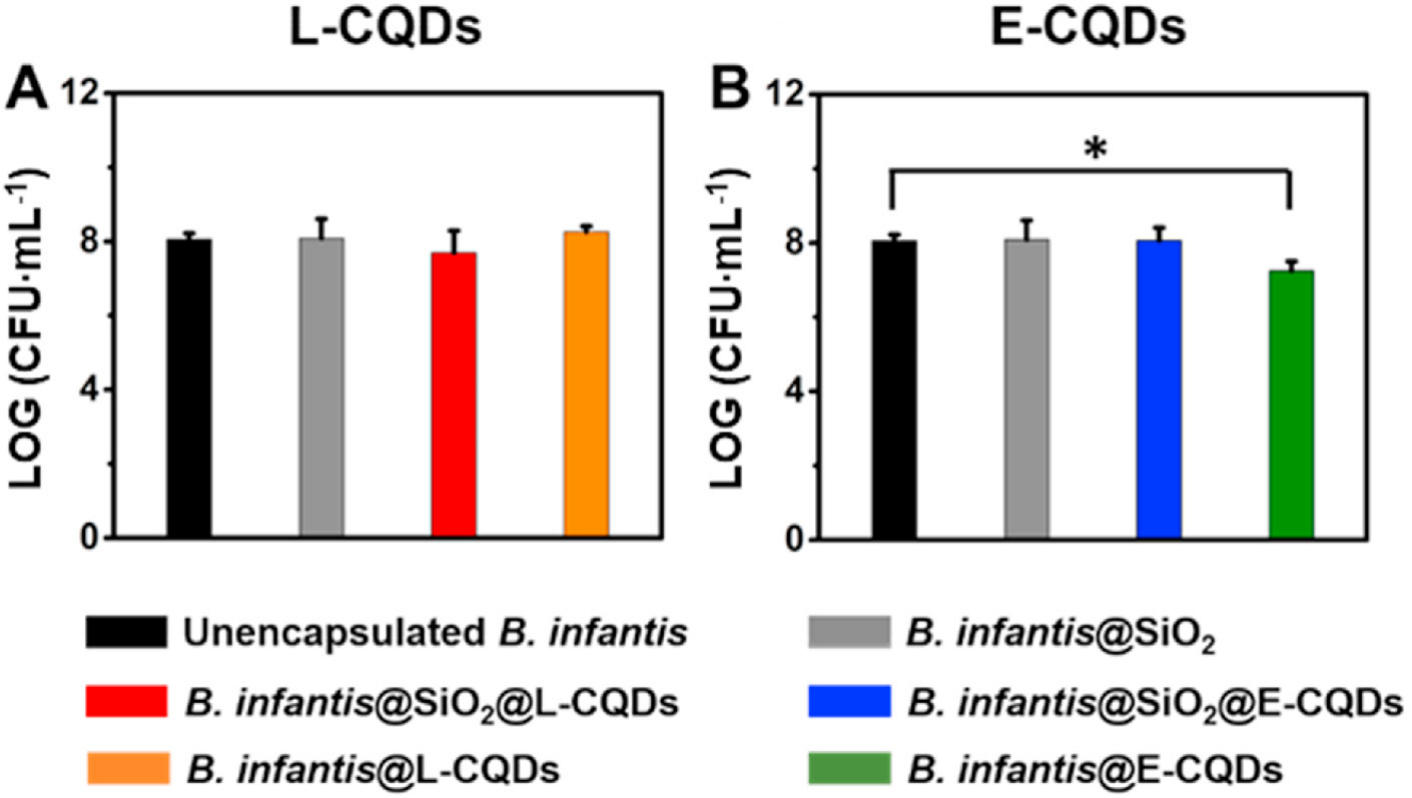
Viability of differently encapsulated *B. infantis* ATCC 15697. **(A)** The number of CFU mL^−1^ of unencapsulated *B. infantis*, *B. infantis* encapsulated in a mesoporous silica nanoparticle layer in absence (*B. infantis*@SiO_2_) or presence of attached *L. acidophilus*-derived CQDs (L-CQDs) or with directly attached CQDs (*B. infantis*@L-CQDs). **(B)** Same as panel A, now for CQDs derived from *E. coli* ATCC 25922 (E-CQDs). All error bars indicate standard deviations over triplicate experiments with separately cultured bacteria. Statistically significant differences (p ​< ​0.05, Student t-test) with respect to unencapsulated *B. infantis* are indicated by spanning bars with an asterisk.

Viability of *B. infantis* after different encapsulations was determined by agar plating and enumeration of the number of colonies forming units (CFUs). Generally, viability of *B. infantis* was not affected by any of the encapsulations applied (Fig. 4). Note however, that direct attachment of E-CQDs in absence of a mesoporous silica nanoparticle layer (Fig. 4B) yielded an extremely minor, statistically significant but microbiologically meaningless reduction of less than 1 log-unit in *B. infantis* viability.

### 3.3 Protection offered by encapsulation towards B. infantis against simulated gastric fluid

**Fig. 5:**
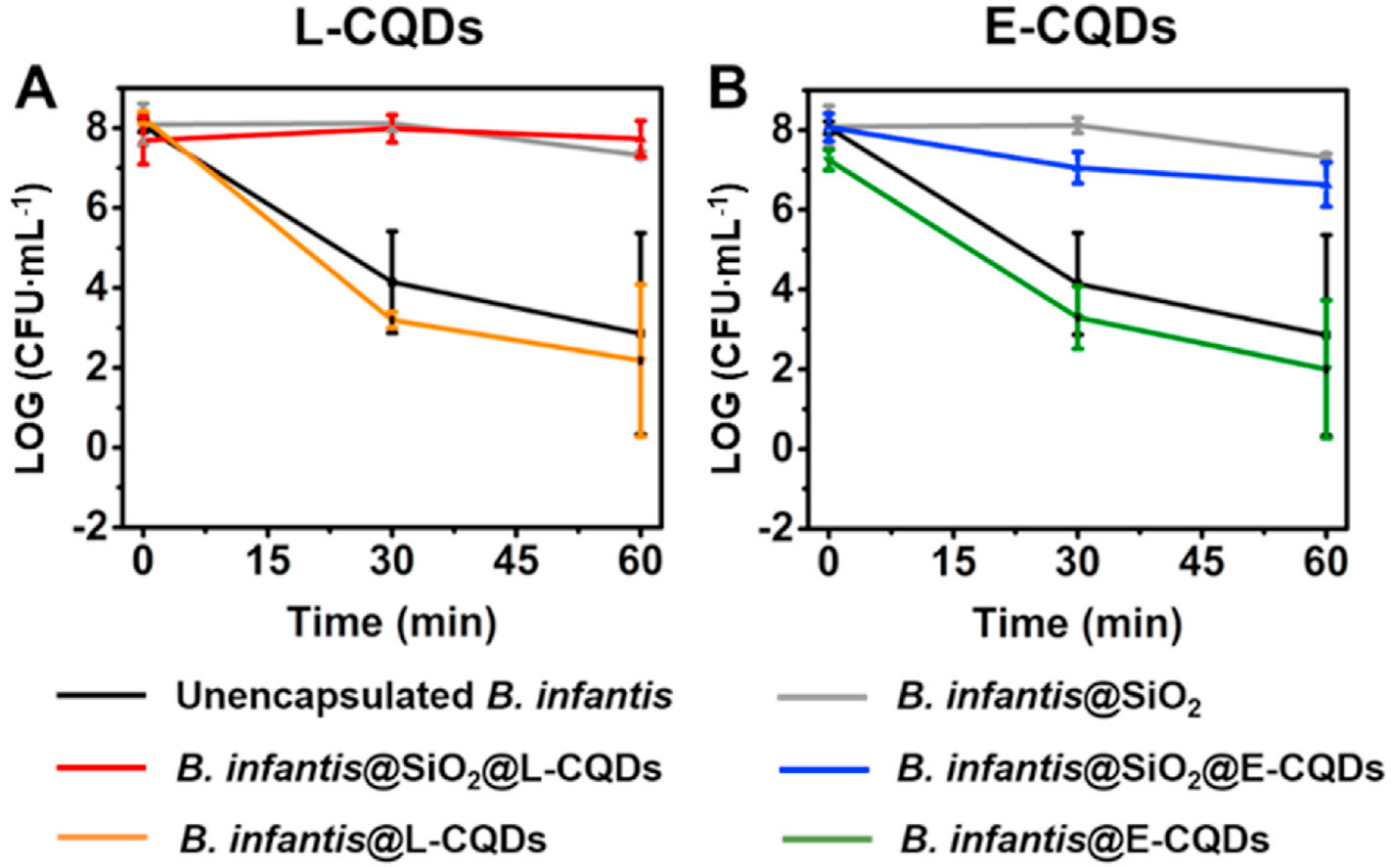
Protection offered by encapsulation towards *B. infantis* ATCC 15697 against simulated gastric fluid (pH 3). **(A)** The number of surviving *B. infantis* as a function of time during exposure up to 60 ​min to simulated gastric fluid for unencapsulated *B. infantis*, *B. infantis* encapsulated in a mesoporous silica nanoparticle layer in absence (*B. infantis*@SiO_2_) or presence of attached *L. acidophilus*-derived CQDs (L-CQDs) or with directly attached CQDs (*B. infantis*@L-CQDs). **(B)** Same as panel A, now for CQDs derived from *E. coli* ATCC 25922 (E-CQDs). All error bars indicate standard deviations over triplicate experiments with separately cultured bacteria.

The protection against exposure to simulated gastric fluid offered by encapsulation in a mesoporous silica nanoparticle shell with attached bacterially-derived CQDs was solely due the mesoporous silica nanoparticle layer and not to attached CQDs (Fig. 5). After 1 ​h exposure, the number of CFUs had decreased by 5 log-units when unencapsulated and in presence of directly attached CQDs, regardless of whether derived from *L. acidophilus* (Fig. 5A) or *E. coli* (Fig. 5B). Oppositely, when encapsulated by a mesoporous silica nanoparticle shell, no decreases in CFUs were observed.

### 3.4 Probiotic activity and reactive oxygen generation by encapsulated B. infantis

**Fig. 6:**
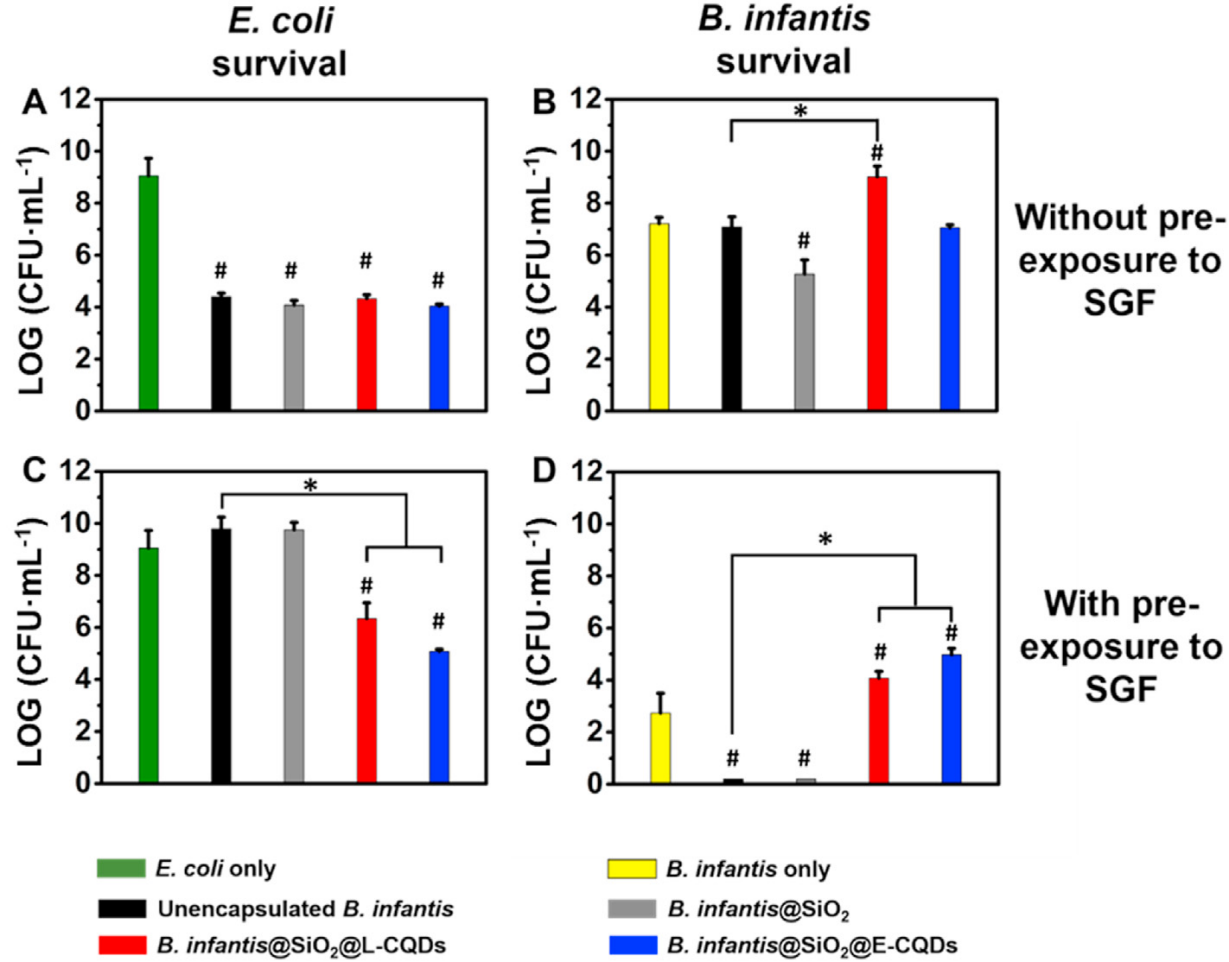
Killing of *E. coli* ATCC 25922 and survival of differently encapsulated *B. infantis* ATCC 15697 in planktonic co-cultures, grown for 24 ​h. **(A)** Number of *E. coli* CFUs after co-culturing with unencapsulated *B. infantis*, *B. infantis* encapsulated in a mesoporous silica nanoparticle layer in absence (*B. infantis*@SiO_2_) or presence of attached *L. acidophilus*- (L-CQDs) or *E. coli* derived CQDs (E-CQDs). **(B)** Number of differently encapsulated *B. infantis* surviving after co-culturing with *E. coli*. **(C)** Same as panel A, now for *B. infantis* pre-exposed to simulated gastric fluid (SGF) for 30 ​min prior to an experiment. **(D)** Same as panel B, now for *B. infantis* pre-exposed to SGF. All error bars indicate standard deviations over triplicate experiments with separately cultured and encapsulated bacteria. Statistically significant differences (p ​< ​0.05, Student t-test) with respect to *E. coli* only, i.e. in absence of probiotic bacteria or with respect to *B. infantis* only, i.e. unencapsulated *B. infantis* without presence of *E. coli*, are indicated by hash-decks, while differences with respect to unencapsulated *B. infantis* are indicated by spanning bars with asterisks.

Probiotic activity by differently encapsulated *B. infantis* was evaluated by planktonically co-culturing with *E. coli*. Co-culturing of *E. coli* with *B. infantis* decreased the number of *E. coli* CFUs significantly by 5 log-units, regardless of whether *B. infantis* was encapsulated or not (Fig. 6A). Alternatively, enhanced *B. infantis* survival was only observed when *L. acidophilus*-derived CQDs were attached to the mesoporous silica nanoparticle layer (Fig. 6B). When pre-exposed to simulated gastric fluid, only *B. infantis* encapsulated with mesoporous silica nanoparticles and bacterially-derived CQDs remained able to kill *E. coli* in significant numbers (Fig. 6C). At the same time, only *B. infantis* encapsulated in a silica nanoparticle layer with attached CQDs survived co-culturing with *E. coli* (Fig. 6D).

Unencapsulated *B. infantis* produced similar amounts of Reactive Oxygen Species (ROS) as did *B. infantis* encapsulated in a mesoporous silica nanoparticle layer (Table 3). Additional attachment of bacterially-derived CQDs activated the silica nanoparticle shell and significantly increased ROS generation, most notably by attachment of CQDs, derived from lactobacilli. This attests to the presence of CQDs also inside the layer.

**Table 3:** ROS generation by unencapsulated *B. infantis*, *B. infantis* encapsulated in a mesoporous silica nanoparticle layer in absence (*B. infantis*@SiO_2_) or presence of attached *L. acidophilus*- (L-CQDs) or *E. coli*-derived CQDs (E-CQDs). ROS generation was measured using oxidation of 2′,7′-dichlorohydrofluorescein (DCFH) into fluorescent 2′,7′-dichlorofluorescein (DCF). All data are expressed in arbitrary units as means ​± ​standard deviations over triplicate experiments with separately cultured and encapsulated bacteria.

Samples	ROS generation (a.u.)
Unencapsulated *B. infantis*	11.7 ​± ​0.7
*B. infantis*@SiO_2_	13.0 ​± ​3.4
*B. infantis*@SiO_2_@L-CQDs	43.7 ​± ​5.5
*B. infantis*@SiO_2_@E-CQDs	32.5 ​± ​2.0

### 3.5 Antibiotic treatment of E. coli colonized epithelial cell layers assisted by encapsulated B. infantis

**Fig. 7:**
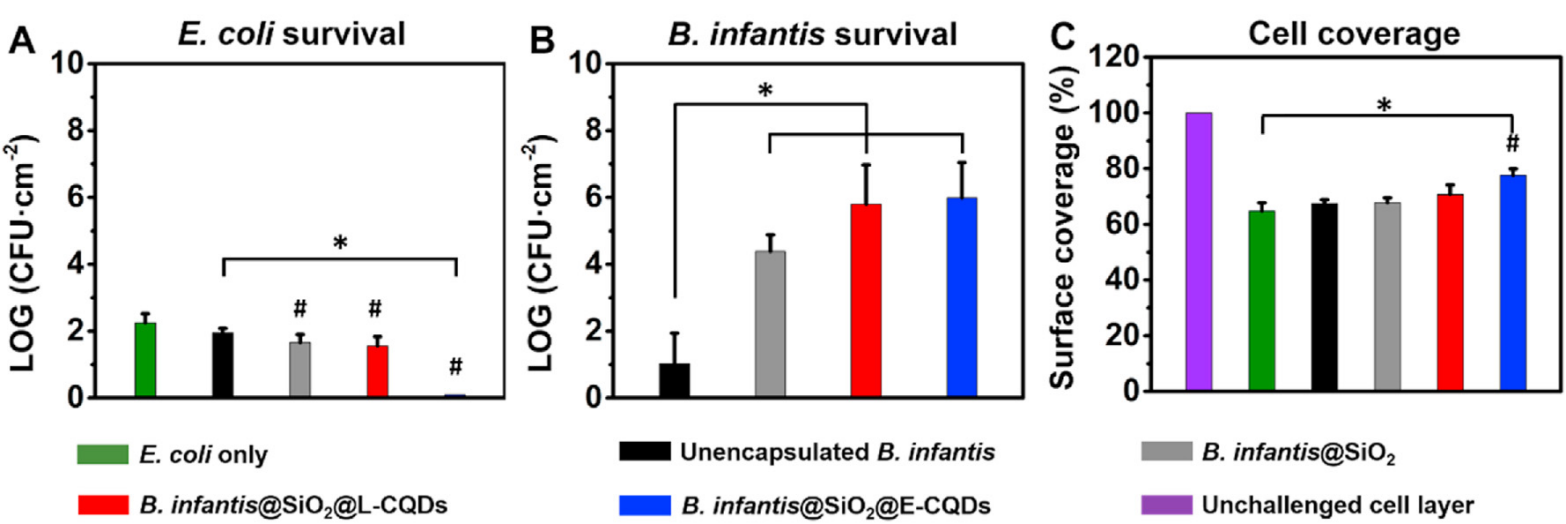
Antibiotic treatment of *E. coli* colonized epithelial cell layers assisted by encapsulated *B. infantis.* Intestinal epithelial layers were colonized during 2 ​h by E*. coli* ATCC 25922 and subsequently co-cultured for 3 ​h in presence of tetracycline (10 ​μg ​mL^−1^) assisted by differently encapsulated *B. infantis*. **(A)** Number of *E. coli* CFUs adhering to intestinal epithelial layers after tetracycline treatment only or assisted by unencapsulated *B. infantis*, *B. infantis* encapsulated in a mesoporous silica nanoparticle layer in absence (*B. infantis*@SiO_2_) or presence of attached CQDs derived from *L. acidophilus* ATCC 4356 (L-CQDs) or *E. coli* ATCC 25922 (E-CQDs). **(B)** Same as panel A, now for the number of *B. infantis* CFUs adhering to intestinal epithelial layers. **(C)** Surface coverage of a transwell membrane by intestinal epithelial cell layers colonized by *E. coli* after *B. infantis* assisted tetracycline treatment. The surface coverage of a cell layer in absence of *E. coli* or *B. infantis* assisted tetracycline treatment was set at 100%. All error bars indicate standard deviations over triplicate experiments with separately cultured cells and bacteria. Statistically significant differences (p ​< ​0.05, Student t-test) are indicated by spanning bars with asterisks, while differences with respect to *E. coli* only, i.e. in absence of probiotic bacteria, are indicated by hash-decks.

Next, differently encapsulated *B. infantis* were pre-exposed to simulated gastric fluid (pH 3) and used to assist tetracycline treatment of an intestinal epithelial layer on a transwell membrane, colonized by *E. coli* (Fig. 7). Colonizing *E. coli* could only be fully eradicated by *B. infantis* encapsulated by a mesoporous silica nanoparticle layer with attached *E. coli* CQDs. At the same time, viable *B. infantis* were found in the highest number after treatment in case of encapsulation in a silica nanoparticle layer with attached bacterially-derived CQDs. A net positive effect on surface coverage by intestinal epithelial cells of tetracycline treatment assisted with encapsulated *B. infantis* could be observed for encapsulated *B. infantis* with attached bacterially-derived CQDs, that was only statistically significant for *E. coli* derived CQDs.

## 4 Discussion

In this paper we developed an active encapsulating layer of boron hydroxyl-modified, mesoporous silica nanoparticles with attached and internalized bacterially-derived CQDs. Bacterial encapsulation in a passive shell of silica nanoparticles to protect bacteria has been described by many others [7,8,26,27]. In our study, the mesoporous silica nanoparticle shell ensured protection of probiotic *B. infantis* against simulated gastric acid and an antibiotic (tetracycline). This type of protection is based on reduced diffusion across the shell and therewith temporary and dependent on pH and antibiotic concentration. Lower pH and higher antibiotic concentration will demonstrate a shorter breakdown of the protective properties of the mesoporous shell. Protection can be enhanced however by creating smaller pore sizes of between 2 and 9 ​nm [9,16], but this goes at the expense of vital exchange processes, negatively affecting viability and probiotic activity. The passive, mesoporous silica nanoparticle shell could be activated here to enhance probiotic activity by the attachment of bacterially-derived CQDs. Activation by attachment of bacterially-derived CQDs yielded more ROS generation by encapsulated *B. infantis* than unencapsulated *B. infantis* or *B. infantis* with only a passive silica nanoparticle shell. Moreover, an active encapsulation with *E. coli* derived CQDs enhanced adhesion and survival on intestinal epithelial layers colonized by *E. coli* during tetracycline treatment (Fig. 7B). These observations proof our hypothesis that active encapsulation of probiotic *B. infantis* by a passive mesoporous silica nanoparticle shell activated through attachment of CQDs derived from pyrolytically carbonized *L. acidophilus* or *E. coli* yields simultaneous protection and enhanced probiotic functionality.

Enhanced *E. coli* killing was demonstrated in planktonic co-cultures of differently encapsulated *B. infantis* and roughly similar when activating CQDs derived from *L. acidophilus* or *E. coli* were attached to the passive silica nanoparticle shell (Fig. 6). However, in a transwell system, only *B. infantis* with an active encapsulation with attached *E. coli* derived CQDs enhanced killing of *E. coli* adhering to intestinal epithelial cell layers, with a positive effect on epithelial cell coverage of the transwell membrane. Attachment of *L. acidophilus* derived CQDs to the passive silica nanoparticle encapsulation did not significantly improve probiotic killing of adhering *E. coli*. Probiotic killing of adhering *E. coli* by *B. infantis* encapsulated in an active shell is an interplay of ROS generation and their ability to adhere to the colonized epithelial layer, because ROS is short-lived [28], requiring generation close to the adhering, target *E. coli*. Encapsulating silica nanoparticle layers activated by *L. acidophilus* derived CQDs generated more ROS than when activated by *E. coli* derived CQDs (Table 3). However, *L. acidophilus* derived CQDs yielded considerably more negatively charged CQDs and encapsulated *B. infantis* than *B. infantis* encapsulated in a silica nanoparticle layer with attached *E. coli* derived CQDs (Fig. 2). Considering that intestinal epithelial cells also possess a negatively charged surface [29], *B. infantis* encapsulated in a shell with attached *E. coli* derived CQDs will experience less electrostatic double-layer repulsion and adhere better through attractive Lifshitz-Van der Waals attraction to intestinal epithelial cell layers than when *L. acidophilus* derived CQDs are employed to activate the shell. Data thus show (Fig. 7A) that the net effect of the interplay between enhanced ROS generation and increased adhesion, is in favor of E-CQDs, i.e. in this interplay increased adhesion is a more important factor than enhanced ROS generation.

Attachment of bacterially-derived CQDs activated a passive, protective shell not only by enhancing adhesive properties but also by enhanced ROS generation of the shell. Previously described active encapsulation of probiotic *Bacillus coagulans* in a layered shell of alginate and chitosan [7] has been described to yield enhanced adhesion to intestinal epithelial cells, but did not enhance ROS generation. Enhanced ROS generation by our CQD-activated mesoporous silica nanoparticle shell is instantaneous and due to the possession of CQDs in and on the mesoporous nanoparticle shell. In absence of instantaneous ROS generation by a shell, encapsulated bacteria first have to grow and break through the shell to exert their probiotic activity [9]. This delays probiotic activity [7]. Other examples of active shells include encapsulation of bacteria in a shell composed of magnetic nanoparticles that can be directed towards a target site using an applied magnetic field [30] or encapsulation in a photocatalytic shell of *E. coli* [31] and *Clostridium pasteurianum* [32] to enhance hydrogen production. The active shell described in our work is unique however, in the sense that it not only yields passive protection to probiotic bacteria, but also activation of both ROS generation and enhanced adhesion. Moreover, since mesoporous silica nanoparticle shells have been shown to allow bacterial growth and breakthrough of off-spring through such shells [16], viable off-spring will be able to colonize the intestinal epithelium to exert their natural probiotic efficacy. Therewith, it bears potential for future clinical application in probiotic-assisted antibiotic-treatment in the fight against antibiotic-resistant bacterial infections.

## 5 Conclusion

We have developed a hybrid probiotic, composed of a probiotic bacterial strain (*B. infantis*) encapsulated in an active mesoporous silica nanoparticle layer with attached bacterially-derived CQDs. The passive mesoporous silica nanoparticle layer protected *B. infantis* against simulated gastric acid and tetracycline, while bacterially-derived CQDs attached to and internalized in the shell activated generation of ROS. The hybrid probiotic demonstrated enhanced antibiotic-assisted *E. coli* killing and probiotic adhesion and survival on intestinal epithelial layers.

Herewith, this work can be considered as a first step towards the development of a fully engineered, probiotic nanoparticle. The first step described here, involves surface-engineering of an active, protective shell with advanced features for enhancing the probiotic functionality of probiotic bacteria. In a next step however, the encapsulating shell might serve as a container for a non-living, chemically active interior, as in artificial cellular bioreactors [33,34]. For the development of fully engineered probiotic nanoparticles, the chemically active interior could be composed of cascade-reaction components [35], producing antimicrobials from endogenously present substances in the human body.

## Credit author statement

**Hao Wei**: Conceptualization, Project administration, Investigation, Validation, Methodology Writing – original draft, Writing – review & editing. **Wei Geng**: Methodology, Writing – review & editing. **Xiao-Yu Yang**: Conceptualization, Writing – review & editing. **Jeroen Kuipers**: Investigation. **Henny C. van der Mei**: Supervision, Conceptualization, Resources, Writing – review & editing. **Henk J. Busscher:** Supervision, Conceptualization, Resources, Writing – review & editing.

## Data availability

Data is available upon request of the authors.

## Declaration of competing interest

The authors declare that they have no known competing financial interests or personal relationships that could have appeared to influence the work reported in this paper.
