# Supplementary material for: Activation of a passive, mesoporous silica nanoparticle layer through attachment of bacterially-derived carbon-quantum-dots for protection and functional enhancement of probiotics
Source: Mater Today Bio. 2022 May 17;15:100293. doi: 10.1016/j.mtbio.2022.100293 (PMC9130534; doi:10.1016/j.mtbio.2022.100293)
Supplement: Multimedia component 1 [file mmc1.docx]

Supporting Information

Activation of a passive, mesoporous silica nanoparticle layer through attachment of bacterially-derived carbon-quantum-dots for protection and functional enhancement of probiotics

Hao Wei, Wei Geng, Xiao-Yu Yang, Jeroen Kuipers, Henny C. van der Mei* and Henk J. Busscher*

Table S1

Decomposition of the N_1s_ photoelectron binding peak of unencapsulated *B. infantis*, *B. infantis* encapsulated with mesoporous silica nanoparticles in absence (*B. infantis*@SiO_2_) or presence of attached, bacterially-derived CQDs (L- or E-CQDs), obtained by XPS.

| **Samples** | **Elemental surface compostions (at%)** | |
| --- | --- | --- |
|  | N_399.8 eV_ (C-NH_2_) | N_401.3 eV_ (C-NH_3_^+^) |
| Unencapsulated *B. infantis* | 1.8 | 0.1 |
| *B. infantis*@SiO_2_ | 2.1 | 0.7 |
| *B. infantis*@SiO_2_@L-CQDs | 2.1 | 0.8 |
| L-CQDs | 5.2 | 0.2 |
| *B. infantis*@SiO_2_@E-CQDs | 2.5 | 1.0 |
| E-CQDs | 2.5 | 0.5 |


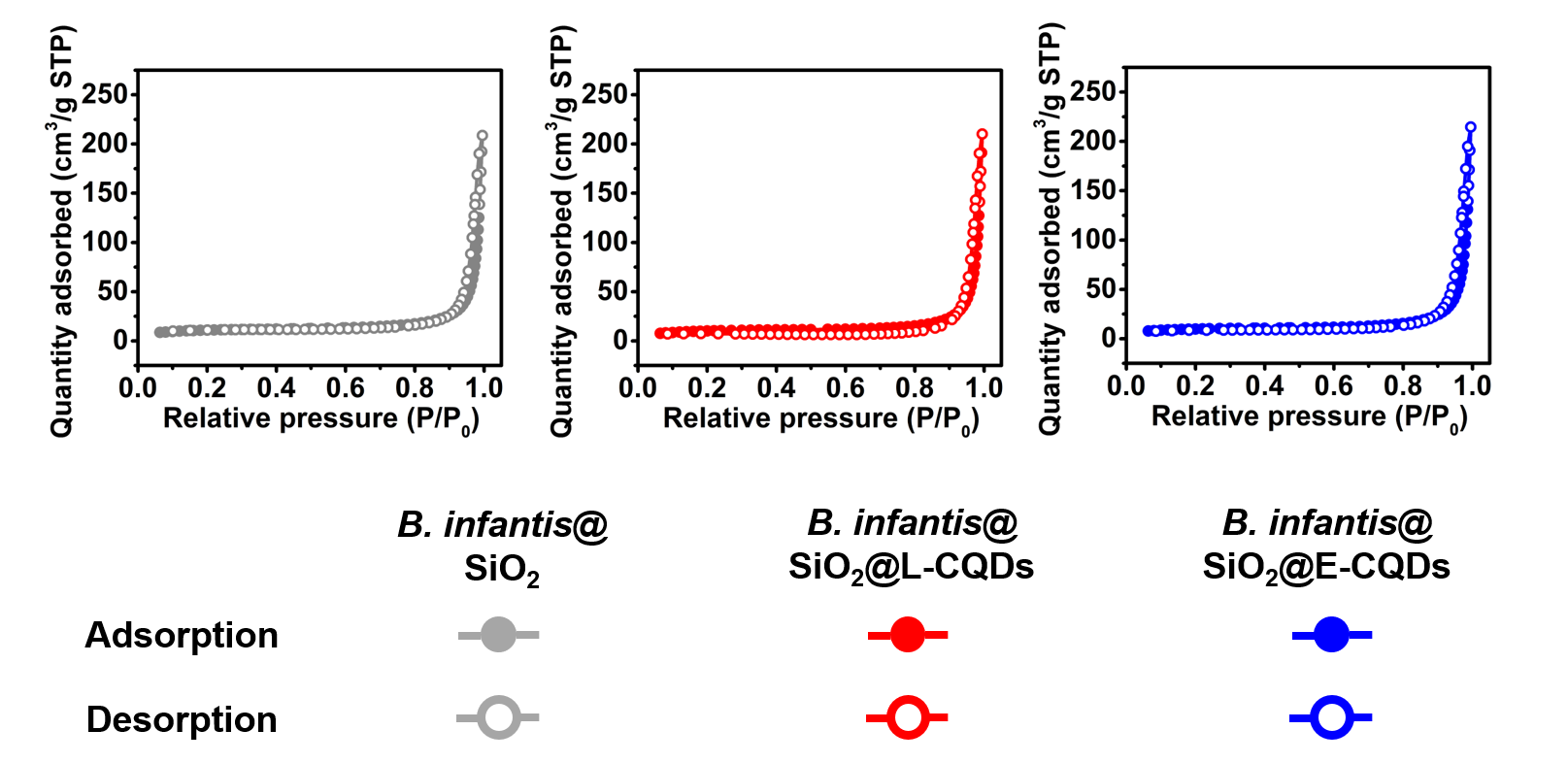


Fig. S1. Nitrogen adsorption/desorption isotherms of *B. infantis* ATCC 15697 encapsulated in a mesoporous silica nanoparticle layer in absence (*B. infantis*@SiO_2_) or presence of attached CQDs (*B. infantis*@SiO_2_@L-CQDs), derived from *L. acidophilus* ATCC 4356 (L-CQDs) or *E. coli* ATCC 25922 (E-CQDs).
